# Extracellular Vesicles as a Potential Therapy for Neonatal Conditions: State of the Art and Challenges in Clinical Translation

**DOI:** 10.3390/pharmaceutics11080404

**Published:** 2019-08-11

**Authors:** Andreea C. Matei, Lina Antounians, Augusto Zani

**Affiliations:** 1 Developmental and Stem Cell Biology Program, Peter Gilgan Centre for Research and Learning, The Hospital for Sick Children, Toronto, ON M5G 0A4, Canada; 2 Division of General and Thoracic Surgery, The Hospital for Sick Children, Toronto, ON M5G 1X8, Canada

**Keywords:** exosomes, hypoxic ischemic encephalopathy, HIE, necrotizing enterocolitis, NEC, retinopathy of prematurity, ROP, bronchopulmonary dysplasia, BPD, spina bifida

## Abstract

Despite advances in intensive care, several neonatal conditions typically due to prematurity affect vital organs and are associated with high mortality and long-term morbidities. Current treatment strategies for these babies are only partially successful or are effective only in selected patients. Regenerative medicine has been shown to be a promising option for these conditions at an experimental level, but still warrants further exploration for the development of optimal treatment. Although stem cell-based therapy has emerged as a treatment option, studies have shown that it is associated with potential risks and hazards, especially in the fragile population of babies. Recently, extracellular vesicles (EVs) have emerged as an attractive therapeutic alternative that holds great regenerative potential and is cell-free. EVs are nanosized particles endogenously produced by cells that mediate intercellular communication through the transfer of their cargo. Currently, EVs are garnering considerable attention as they are the key effectors of stem cell paracrine signaling and can epigenetically regulate target cell genes through the release of RNA species, such as microRNA. Herein, we review the emerging literature on the therapeutic potential of EVs derived from different sources for the treatment of neonatal conditions that affect the brain, retinas, spine, lungs, and intestines and discuss the challenges for the translation of EVs into clinical practice.

## 1. Introduction

According to the World Health Organization, one of the leading causes of pediatric mortality globally is prematurity [1]. With improvements in neonatal intensive care management over the last decades, the number of premature babies, i.e., babies born before 37 weeks of gestation, has been increasing, with an estimated 15 million premature babies worldwide every year [2]. As more premature babies are surviving, we have observed an increase in acute and chronic conditions that are related partly to organ underdevelopment and partly to the invasiveness of supportive measures. Disorders of premature babies that require medical treatment commonly affect the central nervous system, the cardiorespiratory system, and the gastrointestinal tract. These include conditions such as intraventricular hemorrhage (IVH), cerebral palsy, bronchopulmonary dysplasia (BPD), and necrotizing enterocolitis (NEC) [3]. Efforts to cure these diseases have had modest success, and better therapeutic interventions to improve infant morbidity and mortality are called for. Stem cells have surfaced as promising therapies for some of these neonatal conditions, and there has been a large number of experimental studies that have successfully shown improvements in neonatal disease models [4,5,6,7,8]. For some neonatal conditions, such as BPD, favorable experimental findings have been translated to clinical trials in human babies. However, these clinical trials are still ongoing, and it has become clear that stem cell therapy in neonates is challenging. Recognized risk factors and hazards associated with stem cell-based therapy include rejection, toxicity, unwanted biological effects, tumorigenic potential, and contamination by adventitious agents [9]. To overcome these challenges, researchers have investigated whether stem cell derivatives could hold the same regenerative potential as their parent cells without the mentioned associated risks and whether they could be used as alternative therapeutic agents for neonatal conditions. This is also supported by the knowledge that stem cells mainly exert their effects through a paracrine mechanism, whereby their secreted factors signal to target cells.

Recently, extracellular vesicles (EVs) have been identified as one of the key mediators of stem cell paracrine signaling [10], with an effect that, if concentrated, can be greater than that of parent cells [11,12]. EVs are particles that are naturally released by cells, are delimited by a phospholipid bilayer, and do not replicate, as they do not possess a functional nucleus [13]. EVs play a role in intercellular communication by delivering their cargo contents in the form of proteins, lipids, and nucleic acids [13,14,15]. In particular, EV cargo in the form of RNA species have been shown to epigenetically regulate the genes of target cells [16]. Although EVs were described in the literature more than 50 years ago [17], their therapeutic potential has only recently been recognized. Clinical applications of EVs include, on the one hand, EVs as drug carriers, whereby they are used as pharmacological delivery systems for molecules of interests, and on the other hand, EVs as substitutes for stem cell-based therapy for tissue and organ regeneration. Herein, we review the current literature on the therapeutic potential of EVs that are being tested as alternatives to drugs or stem cells as a therapy for neonates. Moreover, we discuss the challenges related to the translation of EVs as therapeutics into clinical practice for this fragile population of patients.

## 2. Methods

A review of the literature was performed using a defined strategy. Searching scientific databases (PubMed, Medline, Scopus), we reviewed studies published in the literature (in English) from 1980 to present that reported the effects of EV treatment on models of neonatal diseases (see Section 8).

For this review, we use the generic term “extracellular vesicles”, or EVs, as endorsed by the International Society for Extracellular Vesicles in their 2018 position statement [13]. However, in descriptions of specific studies, we decided to maintain the same terminology used by their authors, which includes terms such as exosomes.

## 3. Hypoxic Ischemic Encephalopathy

Neonatal hypoxic ischemic encephalopathy (HIE) is a form of brain injury that typically occurs in premature babies and is due to perinatal oxygen deprivation [18,19]. This brain injury leads to damage in cell populations, such as the highly vulnerable immature oligodendrocytes that provide structural support to the brain [20,21]. HIE leads to unfavorable neurodevelopmental outcomes, such as cognitive disorders, in 20–50% of cases, and motor deficits, such as cerebral palsy, in 5–10% of cases [22,23,24,25]. Despite advances that have shown therapeutic benefits using brain cooling, moderate hypothermia is still associated with poor neurodevelopmental outcomes in a proportion of babies, and novel therapeutic strategies are being explored [26]. Researchers initially studied the regenerative potential of stem cell therapies for neuroprotection and repair in experimental models of HIE. Several studies showed that the administration of bone marrow-derived mesenchymal stem cells (BM-MSCs) to neonatal rat pups with hypoxic ischemic injury improved neurologic performance, increased cerebral cell proliferation and differentiation toward neurons and oligodendrocytes, decreased the degree of neuroinflammation, and restored hemispheric volume and axonal connectivity [6,27,28]. Similarly, the administration of MSCs derived from human umbilical cord blood attenuated the severity of brain injury and increased animal survival [29,30]. These results were replicated in an ovine model of hypoxic ischemic injury, where BM-MSCs increased myelination and reduced microglial proliferation, white matter injury, and oligodendrocyte loss [31]. To elucidate the BM-MSC mechanism of action, the same group of researchers tested the therapeutic efficacy of the BM-MSC secretome. This group was the first to report that antenatal administration of EVs derived from BM-MSCs resulted in neuroprotection in an ovine model of hypoxic ischemic injury [32]. This included a reduction in seizure activity and the restoration of subcortical white matter myelination. However, in that study, the authors reported that BM-MSC EVs did not protect against hypoxic ischemic-induced neuroinflammation. In search of another possible mechanism for EV-mediated neuroprotection, the same research group studied whether the administration of BM-MSC EVs could restore blood–brain barrier (BBB) integrity [33]. The BBB is a protective, semipermeable, and highly selective barrier between the brain and systemic circulation. The BBB can be disrupted during HIE, allowing immune cells to enter the central nervous system and induce a neuroinflammatory response [34,35]. Gussenhoven et al. showed that the administration of BM-MSC EVs prevented BBB leakage in fetal ovine brains by targeting the Annexin A1/formyl peptide receptor axis [33]. The BM-MSC EVs were reported to contain Annexin A1, an essential regulator of BBB integrity (Section 8). To prove that the Annexin A1 contained in EVs played a key role, the authors administered purified human Annexin A1 or BM-MSC EVs and confirmed that both improved BBB integrity, which in turn was abolished by the Annexin A1 receptor blocker, the formyl peptide receptor inhibitor [33]. Taken together, these findings suggest the delivery of Annexin A1 via EVs maintains BBB integrity in the immature brain following hypoxic ischemic brain injury. Although the authors did not report the size of these EVs, it is likely the positive effect on the BBB was produced by microvesicles. In fact, it has been recently shown that Annexin A1 is a specific marker of microvesicles shed from the plasma membrane and that Annexin A1-positive vesicles have a size distribution of 150 nm to 1 μm, which is consistent with microvesicles [36].

In 2018, Joerger-Messerli et al. further investigated the neuroprotective effects of another source of MSC-EVs derived from human umbilical cord Wharton’s jelly MSCs (WJ-MSCs) [37]. In this study, the authors reported that the administration of WJ-MSC-EVs to an in vitro model of oxygen–glucose deprivation/reoxygenation in the mouse neuroblastoma cell line neuro2a reduced hypoxic ischemic-induced apoptosis [37]. Joerger-Messerli et al. hypothesized that the effects were mediated by the WJ-MSC EV RNA cargo content, specifically microRNAs (miRNAs), which are noncoding regulatory sequences that regulate target cell gene expression. They showed that fluorescently labeled EV RNA was delivered into neuro2a cells and found that the antiapoptotic effect was likely mediated by the transfer of let-7-5p miRNA from EVs to neuronal cells (Section 8) [37]. Exploring other routes of administration, Sisa et al. tested the intranasal route of BM-MSC EV delivery in experimental hypoxic ischemic injury [26]. In this study, the authors confirmed the beneficial effect of BM-MSC EV administration in reducing microglial activation, cell death, and brain tissue loss.

## 4. Retinopathy of Prematurity

Retinopathy of prematurity (ROP) is a proliferative retinal vascular disease affecting preterm infants and remains the second leading cause of childhood blindness in the United States [38,39]. In preterm infants, retinal development is incomplete, and the degree of retinal immaturity depends on the degree of prematurity. The greatest risk factors for ROP include low gestational age, low birth weight, and supplemental oxygen use [40]. The pathophysiology of ROP is a two-step process, with the first phase occurring when the preterm infant is born and breathes: The retina becomes hyperoxic and levels of vascular endothelial growth factor (VEGF) and insulin-like growth factor 1 (IGF-1) decrease, leading to the cessation of retinal blood vessel growth. Phase two is characterized by disorganized retinal vascular growth and oxidative damage to endothelial cells [39]. Current treatment options for ROP depend on the severity of disease and include laser treatment, bevacizumab, and scleral buckling and/or vitrectomy [39]. Better treatment for ROP is still needed to target aberrant vasoproliferation, facilitate retinal vascular development, and restore ambulatory vision without harmful side effects for the infant.

Promising results have been obtained with stem cell treatment in models of retinal ischemia/reperfusion injury in rats. Li et al. reported that intravitreal injection of BM-MSCs homed to the inner limiting membrane and integrated into the nerve fiber layer and retinal ganglion cell layer [5]. Two to four weeks after transplantation, BM-MSCs differentiated into cell types expressing neuron-specific markers, such as neuron-specific enolase, neurofilament, and neurotrophic factors. Furthermore, BM-MSCs attenuated the reduction of retinal ganglion cells [5]. Dreixler et al. proved that the effect exerted by BM-MSCs could be replicated by intravitreal administration of BM-MSC-conditioned media (CM) in a rat model of retinal ischemia, which restored retinal function and decreased apoptosis [41]. More recently, Moisseiev et al. showed that intravitreal administration of human BM-MSC exosomes preserved retinal vascular flow, reduced neovascularization, and reduced retinal thinning in a mouse model of oxygen-induced retinopathy simulating ROP [42]. Of note, the exosome administration did not provoke an immune response and was not associated with ocular or systemic adverse effects [42]. Proteomic analysis demonstrated that BM-MSC exosomes were packaged with prosurvival-associated proteins, such as those from the cAMP response element-binding protein (CREB) pathway [42]. Aberrant CREB signaling has been associated with retinal ischemia and alterations to the retinal neurotrophic and inflammatory systems, and CREB signaling proteins are critical to the survival of neurons among other cell types [43]. Therefore, intravitreal administration of human-derived BM-MSC exosomes hold therapeutic potential for the treatment of retinal pathologies such as ischemia and ROP.

Further evidence that EV therapy might be beneficial for retinal conditions derives from a recent study that employed microglial-derived exosomes [44]. The rationale behind the use of these exosomes in ROP is that microglial cells play a role in the innate immune response and normal vascular development of the retina [45,46]. Using an in vivo mouse model of oxygen-induced retinopathy, Xu et al. showed that intravitreal administration of microglial-derived exosomes limited the central avascular area, reduced the area of retinal neovascularization, and decreased the expression of hypoxia-induced VEGF, which is considered the most important factor involved in the retinal neovascularization process [44]. Moreover, electroretinography data established better visual function in microglia-derived exosome-treated retinas [44]. RNA sequencing analysis of the EV cargo showed that miR-24-3p levels were high and that this miRNA was shuttled into photoreceptors. Both exosomes and miR-24-3p inhibited the inositol-requiring enzyme 1a (IRE1a)-X-box binding protein 1 (XBP1) pathway, which plays a role in endoplasmic reticulum stress-mediated hypoxia-induced photoreceptor apoptosis [44]. However, miR-24-3p inhibition dramatically reversed this inhibitory effect. Administration of either exosomes or miR-24-3p reduced photoreceptor apoptosis, suggesting that exosomes decreased hypoxia-induced apoptosis by transferring miR-24-3p to target cells. These novel EV-based studies show that different sources of exosomes might be employed as a cell-free treatment for ROP.

## 5. Spina Bifida

Neural tube defects are a group of congenital malformations that result from incomplete neurulation during week four of gestation. Spina bifida is one of the most common and severe forms of neural tube defects and is characterized by an incomplete closure of the spinal column [47]. The most severe subtype of spina bifida is myelomeningocele, which is caused by a failure of the lumbosacral spinal neural tube to close and results in exposure of the spinal cord to toxins and shear stress from amniotic fluid [48]. As a result, infants born with spina bifida often suffer from lifelong paralysis, bowel and bladder dysfunction, and hydrocephalus [49]. Years of experimental research have led to a clinical trial that showed the benefit of antenatal fetal surgical repair via skin closure during the second trimester of pregnancy [50]. This fetal surgical technique decreases the need for cerebrospinal fluid shunting; however, there is little improvement in motor function or the damage to the exposed spinal cord in babies with spina bifida who undergo this surgery [51]. 

Therefore, in search of a regenerative therapy that would be an adjunct to fetal surgical repair for spina bifida, researchers have explored the use of stem cell implantation to allow neuronal regeneration. Li et al. showed that topical administration of BM-MSCs to the spinal column of rats with spina bifida during fetal surgery regenerated neurons and reduced spinal neuron death in the defective spinal cord [7]. For a source of stem cells derived from tissues that are closer to the fetus, the placenta has been shown to be a promising source for human MSCs (P-MSCs) [52]. Studies have demonstrated that P-MSCs are neuroprotective, immunomodulatory, and improve wound healing [53,54,55]. The administration of P-MSCs has been shown to be beneficial in two different models: the first in lambs, where in utero administration of P-MSCs to an ovine model of spina bifida improved limb motor function [56]; and the second in a rat model of spina bifida induced via the administration of retinoic acid, where P-MSC-seeded patches placed in utero had significantly less dense apoptotic cells at the site of injury compared to patches without P-MSCs [51]. These previous studies showed that P-MSCs did not engraft into the host tissue, suggesting they mediate their effects through a paracrine mechanism rather than their direct differentiation [55,57]. To investigate whether P-MSCs mediate their effects via EV release, Kumar et al. reported on a study where they used the staurosporine-induced apoptotic human neuroblastoma cell line, SH-SY5Y [58]. In this study, the authors found that P-MSC exosomes increased the number of neuronal projections, the total number of branch points, circuitry length, and tube length, suggesting they play a role in neuroprotection [58]. To identify the potential mediators of P-MSC-derived EVs, Kumar et al. performed proteomics and an RNA sequencing analysis and found that galectin 1 was highly expressed on the surface of P-MSCs and P-MSC exosomes. Galectin 1 has both immunomodulatory and neuroprotective functions and has been demonstrated to regulate axonal regeneration, cell adhesion, and the proliferation of neural stem cells [59,60]. For further proof that this effect was mediated by galectin 1, Kumar et al. pre-incubated P-MSC exosomes with anti-galectin 1 antibody. Upon administration, a decrease in the total number of branching points and total neurite segments and diminished neuroprotective effects were observed [58]. These encouraging findings initiated the prospect for EV therapeutic application of P-MSC exosomes, either alone or in combination with trophic factors, for the treatment of spina bifida.

## 6. Bronchopulmonary Dysplasia

Bronchopulmonary dysplasia (BPD) is a lung disease, multifactorial in nature, which almost exclusively affects preterm babies [61]. Lungs affected by BPD are immature, as alveolar development and pulmonary angiogenesis are arrested. Moreover, these lungs are susceptible to inflammation, infection, and injury secondary to intensive care maneuvers, such as mechanical ventilation [3]. BPD is a leading cause of significant mortality and morbidity, including long-term respiratory and neurodevelopmental sequelae that extend beyond childhood [62]. Over the years, there has been extensive research aimed at finding an effective therapy for babies with BPD, and promising results have been obtained with the use of MSCs [63]. Experimental evidence has led to the translation of MSC clinical application, so that at present, there are ongoing clinical trials using MSCs in human babies with BPD [64,65,66]. However, several studies have demonstrated that the administration of MSC CM was not only beneficial in preventing alveolar loss, but also had a greater regenerative potential than parent cells did in BPD [4,67,68,69]. In fact, experimental studies have also shown improvements in alveolar loss in the absence of MSC engraftment in the target lungs, thus confirming a paracrine mechanism of action [70].

With this in mind, and given the concerns that MSC administration might induce tumor formation, researchers have started exploring the role of EV-based therapy in experimental BPD [65,71,72]. Lee et al. were the first to report that intravenously administered exosomes derived from BM-MSCs and WJ-MSCs, but not exosome-depleted CM, were able to suppress pulmonary macrophage influx and inhibit pulmonary vascular remodeling, ameliorating pulmonary hypertension [72]. In this study, the authors used adult mice and modeled hypoxia-induced pulmonary hypertension, a form of lung damage similar to BPD. When they investigated the cargo content of BM-MSC exosomes, they found an upregulation of miR-16, miR-21, and let7b pre-miRNA. The same group of researchers more recently compared the efficacy of exosomes derived from BM-MSCs and WJ-MSCs in a mouse model of BPD and showed that both improved lung structure and function [70]. Specifically, exosomes from both sources improved alveolarization and lung angiogenesis and decreased collagen, fibrosis, arteriole muscularization, and pulmonary hypertension [70]. The authors performed pulmonary function tests on mice and showed that exosomes reduced BPD-related increased lung capacity and emphysema [70]. Moreover, Willis et al. reported that the administration of WJ-MSC exosomes to pulmonary macrophages in vitro modulated their phenotype toward M2-like anti-inflammatory macrophages and suppressed M1-like proinflammatory macrophages [70].

Other research groups have shown similar beneficial effects with MSC exosome administration in experimental BPD. Chaubey et al. reported that CM and exosomes derived from human umbilical cord MSCs (hUC-MSCs) isolated from Wharton’s jelly collected at 25–30 weeks of gestation attenuated the degree of BPD in neonatal mice [73]. The authors concluded that the effect of exosomes was partly mediated by tumor necrosis factor alpha-stimulated gene-6 (TSG-6), an anti-inflammatory factor present in hUC-MSCs. Specifically, hUC-MSC exosome intraperitoneal administration reversed lung inflammation, alveolar injury, alveolar–capillary leak, and pulmonary hypertension [74]. When they examined the brains of BPD pups, they found that hUC-MSC exosome-treated mice had less neuronal apoptosis and restored myelination.

Braun et al. also used the intraperitoneal route to administer BM-MSC exosomes in a rat model of BPD [74]. In this study, the authors showed that BM-MSC exosomes preserved alveolar growth, increased peripheral blood vessel formation, and decreased the degree of pulmonary hypertension in experimental BPD. Moreover, this group showed that in an in vitro tube formation assay using human umbilical vein endothelial cells, BM-MSC exosomes promoted angiogenesis in part through a VEGF-mediated mechanism [75]. Similarly, Ahn et al. showed that VEGF mediated the therapeutic efficacy of MSC-derived EVs in a model of neonatal hyperoxic lung injury [75]. In rats with BPD, the authors compared the intratracheal administration of either hUC-MSC exosomes, hUC-MSCs, or hUC-MSC VEGF knockdown exosomes transfected with siRNA [75]. Ahn et al. found that MSCs and MSC-derived EVs, but not EVs derived from VEGF knockdown MSCs, were able to attenuate the degree of cell death, inflammation, and impaired alveolarization and angiogenesis. Importantly, this study showed that hUC-MSC EVs were as effective as parental MSCs at attenuating neonatal hyperoxic lung injuries and that this effect was mediated primarily by the transfer of VEGF [75]. Conversely, Porzionato et al. reported that intratracheal administration of hUC-MSC EVs was more effective than hUC-MSCs in repairing hyperoxia-induced lung injury [12]. In fact, hUC-MSC EVs had the most significant increase in total number of alveoli, decrease in mean alveolar volume, and decrease in arteriole muscularization compared to the hUC-MSCs from which they were derived [12].

## 7. Necrotizing Enterocolitis

Necrotizing enterocolitis (NEC) remains one of the most common and severe gastrointestinal emergencies in the neonatal period, primarily affecting premature neonates and extremely low-birth-weight infants (<1000 g) [76,77]. NEC is characterized by an extensive intestinal inflammatory process, ranging from mucosal injury to full-thickness necrosis and perforation that often leads to systemic inflammation affecting distant organs, including the brain [76,78,79]. The etiology is considered multifactorial, with several contributing causes, such as prematurity, formula feeding, hypoxia, and bacterial contamination [77]. Preterm babies who develop NEC have an immature gastrointestinal tract and naïve immune system that predispose them to the development of NEC [80,81]. Moreover, premature babies are at risk of NEC not only because they are formula-fed, which predisposes them to bowel ischemia, but also because they are not breast-fed, which is recognized as being a protective measure against NEC [76,77]. Despite advancements in the medical and surgical treatment of NEC over the last six decades, mortality is still very high and remains at 30–50%.

Several treatment strategies have been tested in experimental models of NEC, and stem cell therapy has emerged as an attractive treatment option. In a neonatal rat model of NEC, intraperitoneal administration of amniotic fluid stem cells (AFSCs) improved survival and morbidity, decreased NEC incidence, improved intestinal damage and function, decreased bowel inflammation, and regenerated the damaged bowel by increasing enterocyte proliferation and reducing apoptosis [8]. The beneficial effect of AFSCs was achieved via the modulation of stromal cells expressing cyclooxygenase 2 in the lamina propria, as shown by survival studies using selective and nonselective cyclooxygenase 2 inhibitors. In that study, compared to AFSCs, BM-MSC administration was not as effective at improving pup survival [8,82]. Interestingly, the beneficial effect exerted by AFSCs on the damaged neonatal intestine was achieved despite a low degree of cell engraftment, thus suggesting a paracrine mechanism of action. This observation has also been reported by other research groups who used different sources of MSCs, such as the bone marrow and the umbilical cord, and concluded that the administered stem cells were releasing factors that could regenerate the damaged bowel [83,84].

Recent studies have shown that stem cell-derived EVs play a crucial role in paracrine signaling, and researchers are now focusing on EVs as a potential cell-free therapy for NEC babies. Rager et al. were the first to report that the biologically active vectors of BM-MSCs administered to neonatal rats with NEC were the exosomes released by the cells [85]. Intraperitoneal administration of BM-MSC exosomes led to a significantly lower incidence of NEC and improvement in the severity of intestinal injury. BM-MSC exosomes proved to restore the intestinal barrier function to levels comparable to BM-MSC treatment alone [85]. Using an in vitro wound healing assay with intestinal epithelial cells (IEC-6), the authors confirmed that the beneficial effects of BM-MSC exosomes were specific, as they were not replicated by exosome-depleted BM-MSC CM [85]. In a rat model of NEC, the same group of researchers demonstrated a reduction of NEC incidence, employing exosomes derived from different types of stem cells (e.g., amniotic fluid-derived MSCs, BM-MSCs, amniotic fluid-derived neural stem cells, and neonatal enteric neural stem cells) [86]. A decrease in the severity of intestinal injury upon a histological analysis was observed with increasing exosome concentrations [86]. This observation is in line with other reports where EV effects were dose-dependent and not influenced by variations in EV size distribution or the method of isolation [87].

Similar protective effects toward bowel damage in experimental NEC were obtained by other groups with the use of milk-derived EVs. As previously mentioned, breastfeeding is associated with a decreased incidence of NEC; however, the protective mediators against NEC present in breastmilk are only partly known [88]. In 2017, Hock et al. were the first to report that in an in vitro model of bowel damage, exosomes derived from rat breastmilk promoted epithelial cell viability, proliferation, and stem cell activity [89]. Similarly, Martin et al. showed that exosomes derived from human breast milk conferred protection to intestinal epithelial cells from H_2_O_2_-induced oxidative stress [90]. These results were in line with the observation by Chen et al. that porcine milk-derived exosomes increased the villus height and crypt depth of the murine intestine [91]. Wang et al. compared breastmilk-derived exosomes from mothers who delivered term versus preterm babies [92]. The authors demonstrated that preterm exosomes had an enhanced ability to improve the proliferation of intestinal epithelial cells compared to term exosomes [92]. A proteomic analysis showed that the preterm exosomes contained peptides that participated in metabolic, developmental, and immune system processes; biological adhesion; and cell proliferation [92]. 

Due to the scarcity of breastmilk availability, Li et al. explored the role of exosomes in widely available bovine milk in a model of human intestinal cells [93]. Exosome administration promoted goblet cell expression, as confirmed by increased mucin production and increased goblet cell-associated markers (trefoil factor 3 and mucin 2). Goblet cells and their product mucin are known to be impaired in the intestine during NEC [94]. Moreover, Li et al. showed that bovine milk-derived exosomes reduced mucosal inflammation and protected against intestinal injury in neonatal mice with NEC [93]. Taken together, all of these studies call for further exploration into the development of EV-based therapies for the prevention and treatment of NEC.

## 8. Summary of Neonatal Conditions with EV Based Therapy Reported in the Literature 

**Figure 1:**
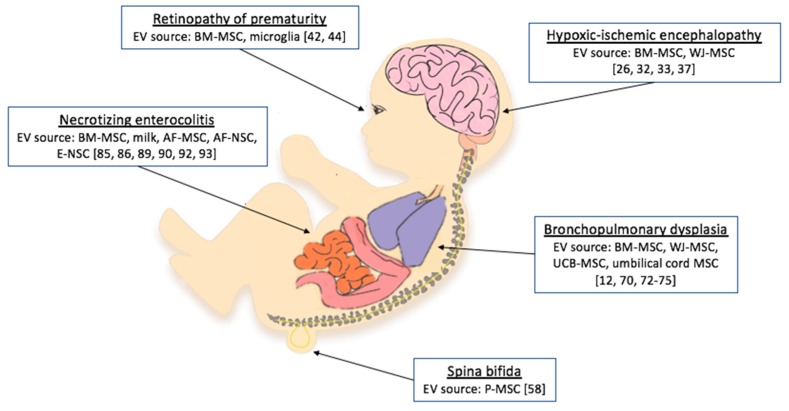
Neonatal conditions for which studies have tested extracellular vesicle (EV) administration as a treatment option. BM-MSCs, bone marrow mesenchymal stem cells; AF-MSCs, amniotic fluid mesenchymal stem cells; AF-NSCs, amniotic fluid neural stem cells; E-NSCs, neonatal enteric neuronal stem cells; P-MSCs, placental mesenchymal stem cells; WJ-MSCs, umbilical cord Wharton’s jelly mesenchymal stem cells; UCB-MSCs, umbilical cord blood-derived mesenchymal stem cells [12,26,32,33,37,42,44,58,70,72,73,74,75,85,86,89,90,92,93].

Here we present all reported effects of EV treatment on models of neonatal diseases (Figure 1). Details on EV species, source, isolation technique, and administration route are reported in Table 1. Moreover, we focused on the mediators of EV beneficial effects and the pathways affected by their cargo, which are ported in Table 2.

## 9. Considerations and Challenges in the Therapeutic Application of EVs: Identity, Potency, Purity, Safety, and Quality

The promising results obtained with EVs in experimental models of neonatal conditions reported here are an encouraging foundation for the translation of EV-based therapies into clinical practice. However, there are several challenges that remain to be overcome. The identity of the EVs remains partially unknown, especially the nature of their therapeutically active components. Efforts are being progressively made to understand the bioactive elements of the EV cargo, as shown in Table 2. EVs are known to contain proteins, lipids, and genetic material, yet there is still a lack of knowledge regarding the role of some noncoding RNA species, such as piwi-interacting RNAs (piRNAs), ribosomal RNAs (rRNAs), small nuclear RNAs (snRNAs), small nucleolar RNAs (snoRNAs), long ncRNAs (lncRNAs), long intergenic RNAs (lincRNAs), and circular RNAs. Similarly, the DNA contained in EVs has an undefined role. Only recently, double-stranded DNA (dsDNA) was shown to be present in EVs, and mutations in parent cells were identified in the EV dsDNA [95]. Studies on patients with pancreatic cancer have shown that EVs from human serum samples contain genomic DNA spanning all chromosomes, indicating their use as biomarkers for genomic mutations in cancer patients [96]. Takahashi et al. proposed that the dsDNA fragments contained in EVs represent in part one way that parent cells maintain homeostasis by removing harmful cytoplasmic DNA [97]. Recently, Jeppesen et al. assessed the composition of small EVs (exosomes) through high-resolution density gradient fractionation and direct immunoaffinity capture and challenged the presence of DNA in small EVs [36]. According to that study, dsDNA is present only in larger EVs, i.e., microvesicles. Hence, the quest for the identity of the bioactive components of EVs continues.

Another important aspect to consider in the use of EVs as therapeutics is their potency. There are several methods to quantify EV doses, and the most common are based on the number of parent cells (cell equivalents), EV protein cargo (protein concentration), and EV number and size using specialized quantitative analytical measurements, such as tunable resistive pulse sensing and nanoparticle tracking analysis [98]. In the studies included in our review, dose, frequency, and route of administration varied considerably, and the safe and effective dose to be used in the treatment of such neonatal conditions has yet to be determined. It has been reported that EVs have a short half-life and that their effects could be short-lived [99,100]. Therefore, to maintain their therapeutic potential over time, researchers have administered EVs in repeat doses [12,101]. Sjoqvist et al. observed that the repeated administration of EVs was more important than the dose itself in promoting wound healing in a pig model of esophageal wound repair [100]. Furthermore, repeat doses have been reported to not result in increased toxicity and immunogenicity [102,103].

Another challenge for the use of EVs as therapy relates to the purity of EV preparations [104]. Purity has been expressed as the ratio between protein content and the number of EVs and has been considered to be directly related to the EV isolation technique [104]. For this reason, several studies have assessed different isolation techniques to improve EV purity and to clear preparations of coprecipitates [104,105,106,107]. Isolation methods are recognized to play a role not only in EV purity, but also in EV yield, size distribution, and potential biological effects, and therefore they are considered crucial in obtaining optimal EV preparation. The different methods currently in use to isolate EVs include differential sedimentation (ultracentrifugation), density gradient, size exclusion chromatography, and kit-based isolation [87,108]. Several studies have shown that different isolation methods yield a different amount of EVs [87,107,109,110,111,112]. Variable yield, i.e., the number of isolated EV particles, has been shown to result in variable biological effects, regardless of the method employed to isolate the EVs [87]. Antounians et al. recently reported that when equal volumes of EVs isolated with different methods were used as a treatment in a model of lung epithelial injury, a significant biological variation was observed [87]. This effect appeared to be dose-dependent, as was also reported by other groups, thus highlighting the importance of choosing the right isolation technique for optimal yield [70,113,114]. To reduce the variability due to different isolation techniques, it has been proposed that the efficacious EV dose could be quantified. This could be obtained through surrogate markers for EVs, such as microRNAs, through fingerprinting assays [98].

The safety of EV preparations is also paramount to their translation into clinical practice. To date, a number of clinical trials have demonstrated the safety and feasibility of an EV-based approach [115]. The International Society for Extracellular Vesicles has published a position paper for applying EV-based therapeutics in clinical trials and has highlighted the safety and regulatory requirements that must be considered for pharmaceutical manufacturing and clinical applications [115]. However, to the best of our knowledge, there have been no reported trials to date that have tested the safety of EVs as a therapy in neonates. For this fragile population of patients, similar stringent regulations would apply, and the potential for an immunological response to treatment would need to be addressed.

Alongside safety considerations, the quality control of EV preparations is critical to ensure that a sustainable EV source is maintained and that variations between batches of EVs are minimized. Furthermore, compliance with regulations outlined by regulatory and scientific bodies should safeguard the production of EVs through good manufacturing practices. Lastly, the scalability of EVs for isolation, administration, and storage should be considered [116]. While the regulatory frameworks for manufacturing and clinical trials already exist, specific guidelines for using EVs as therapeutics are currently being established [115,117,118].

## 10. Final Remarks

Although stem cell-based therapies have emerged as a treatment option for some orphan diseases, in the last few years we have witnessed a paradigm shift toward cell-free therapy. EVs have been shown to be a promising alternative for many different diseases, including those that affect neonates, for whom cell-based therapies might be challenging or hazardous.

As EVs are considered the key mediators of stem cell paracrine signaling, the most common source of EVs used as treatment agents is from stem cells. In particular, the large body of literature on the use of MSCs has helped further the testing of EVs derived from MSCs in a variety of disease models. However, other sources of EVs have recently emerged. For example, milk is a source of EVs that appears to have regenerative potential in neonatal bowel conditions such as NEC.

The explosion of interest in EV-based therapies is still at an experimental stage. Nevertheless, for certain neonatal conditions, such as BPD, there are already a number of preclinical studies from different research groups that are concordantly showing a beneficial effect with EV administration. On the other hand, the use of EVs as treatment for diseases such as ROP and spina bifida is encouraging, but has been limited to a few experimental reports. Further studies are needed to confirm the beneficial effects of EVs in neonatal conditions before these findings can be tested in clinical trials. In addition, little is known about the mechanisms by which EVs exert their effects and the bioactive mediators present in their cargo. Some studies have reported EV effects mediated by proteins, but increasingly the focus has shifted to the RNA species contained inside the EVs and their potential to epigenetically regulate target cells. Lastly, further studies are required to address the possible side effects of EVs and to overcome the challenges related to their therapeutic application. Nonetheless, we believe that innovative EV-based therapies are opening new avenues in neonatal medicine as alternatives to drugs and stem cells.

## Figures and Tables

**Table 1:** Studies reporting the use of EVs as treatment for neonatal conditions.

Study	EV Source	EV Isolation Technique	EV Administration Route	Model	Biological Effect
Hypoxic Ischemic Encephalopathy (HIE)					
Ophelders et al. 2016 [32]	Human BM-MSCs	PEG + NaCl, low-speed centrifugation	In utero intravenous	In vivo: HIE ovine model	Reduced number and duration of seizures, restored myelination, restored baroreflex sensitivity
Joerger-Messerli et al. 2018 [37]	Human WJ-MSCs	Serial centrifugation	Addition into culture media	In vitro: mouse neuroblastoma cell line neuro2a	Protected against hypoxic ischemic-induced apoptosis in neuronal cells
Sisa et al. 2019 [26]	Human BM-MSCs	UC	Intranasal	In vivo: HIE mouse model	Decreased microglia activation, cell death, and tissue loss, and improved behavior
Gussenhoven et al. 2019 [33]	Human BM-MSCs	PEG, low-speed centrifugation	In utero intravenous	In vivo: HIE ovine model	EVs containing AnnexinA1 restored blood–brain barrier integrity
Retinopathy of Prematurity (ROP)					
Moisseiev et al. 2017 [42]	Human BM-MSCs	Tangential flow filtration	Intravitreal	In vivo: ROP mouse model	Preserved retinal blood flow and reduced retinal thickening, decreased severity of retinal ischemia
Xu et al. 2019 [44]	BV2 microglial cells	UC	Intravitreal	In vivo: ROP mouse model	Reduced central avascular area, decreased neovascularization and VEGF, suppressed photoreceptor apoptosis, alleviated ER stress
Spina Bifida (SB)					
Kumar et al. 2019 [58]	Human P-MSCs	Differential centrifugation	Addition into culture media	In vitro: Human neuroblastoma cell line (SH-SY5Y)	Increased number of neurites, exerted neuroprotective effect mediated through Galectin 1
Bronchopulmonary Dysplasia (BPD)					
Lee et al. 2012 [72]	Mouse BM-MSCs and human WJ-MSCs	Ultrafiltration, PEG, size exclusion chromatography, UC	Left jugular vein or tail vein	In vivo: HPH mouse model	Suppressed pulmonary macrophage influx and inhibited pulmonary vascular remodeling
Braun et al. 2018 [74]	Rat BM-MSCs	UC	Intraperitoneal	In vivo: BPD rat model In vitro: Human umbilical vein endothelial cells	Protected alveolarization and angiogenesis Increased capillary network formation via VEGF
Ahn et al. 2018 [75]	Human UCB-MSCs	UC	Intratracheal	In vivo: BPD rat model	EVs promoted alveolarization and angiogenesis, decreased cell death, attenuated macrophages and proinflammatory cytokines via VEGF
Chaubey et al. 2018 [73]	Human WJ-MSCs from mothers delivering preterm babies	Differential centrifugation	Intraperitoneal	In vivo: BPD mouse model	Ameliorated pulmonary inflammation, alveolar–capillary leakage, alveolar simplification, and pulmonary hypertension Improved lung, cardiac, and brain pathology through TSG-6
Willis et al. 2018 [70]	Human WJ-MSCs and human BM-MSCs	Flotation on OptiPrep cushion	Intravenous	In vivo: BPD mouse model In vitro: mouse bone marrow-derived macrophages or alveolar macrophages	Promoted alveolarization and angiogenesis, improved pulmonary function, modulated macrophage phenotype (augmenting anti-inflammatory subtype) Reduced proinflammatory markers and promoted anti-inflammatory markers
Porzionato et al. 2018 [12]	Human umbilical cord MSCs	Tangential flow filtration	Intratracheal	In vivo: BPD rat model	Reduced hyperoxia-induced lung damage, with EVs performing better than parent cells at maintaining alveolarization and lung vascularization
Necrotizing Enterocolitis (NEC)					
Rager et al. 2016 [85]	Murine BM-MSCs	P100 PureExo Exosome Isolation reagent (in vivo) UC (in vitro)	Intraperitoneal	In vivo: NEC rat model In vitro: intestinal epithelial cell (IEC-6) wound healing assay	Decreased incidence and severity of NEC and preserved gut barrier function Improved cell mobility and wound healing
Hock et al. 2017 [89]	Rat breastmilk	ExoQuick reagent	Addition into culture media	In vitro: rat small intestine epithelial cells (IEC-18)	Increased cell proliferation and intestinal stem cell activity
McCulloh et al. 2018 [86]	Rat BM-MSCs, AF-MSCs, AF-NSCs, E-NSCs	UC	Intraperitoneal	In vivo: NEC rat model	All sources of exosomes reduced NEC incidence and severity at a concentration of 4.0 × 10^8^ particles
Martin et al. 2018 [90]	Human breastmilk	UC	Addition into culture media	In vitro: rat small intestine epithelial cell line (IEC-18)	Protected against oxidative stress
Li et al. 2019 [93]	Bovine breastmilk	UC	Orogastric	In vivo: NEC mouse model In vitro: LS174T human colonic cells	Protected ileum from NEC-induced alterations, increased goblet cell expression Promoted goblet cell expression and increased mucin production
Wang et al. 2019 [92]	Human breastmilk from mothers delivering preterm versus term babies	UC	Orogastric	In vivo: NEC rat model In vitro: Human normal intestinal epithelial FHC	Preterm milk exosomes protected villous integrity, restored enterocyte proliferation Preterm milk exosomes improved proliferation of intestinal epithelial cells compared to term milk exosomes 70 significantly modulated peptides, from 28 parent proteins, were differentially expressed in preterm milk exosomes compared to term milk exosomes

Abbreviations: BM-MSCs, bone marrow mesenchymal stem cells; PEG, polyethylene glycol; WJ-MSCs, umbilical cord Wharton’s jelly mesenchymal stem cells; UC, ultracentrifugation; P-MSCs, placental mesenchymal stem cells; VEGF, vascular endothelial growth factor; HPH, hypoxic pulmonary hypertension; UCB-MSCs, umbilical cord blood-derived mesenchymal stem cells; TSG-6, tumor necrosis factor alpha-stimulated gene-6; AF-MSCs, amniotic fluid-derived mesenchymal stem cells; AF-NSCs, amniotic fluid-derived neural stem cells; E-NSCs, neonatal enteric neuronal stem cells; HIE, hypoxic ischemic encephalopathy; ROP, retinopathy of prematurity; SB, spina bifida; BPD, bronchopulmonary dysplasia; NEC, necrotizing enterocolitis.

**Table 2:** Reported mediators of EV beneficial effects.

Cargo Type	Study	EV Source	Disease Model	Factor(s)	Pathways and Biological Functions
Proteins	Moisseiev et al. 2017 [42]	Human BM-MSCs	ROP	cAMP response element-binding protein pathway	Prosurvival heat shock protein pathways
	Braun et al. 2018 [74]	Rat BM-MSCs	BPD	VEGF	Lung vascularization and alveolarization
	Ahn et al. 2018 [75]	Human UCB-MSCs	BPD	VEGF	Lung vascularization and alveolarization, decreased IL-1α, IL-1β, IL- 6, TNF-α
	Chaubey et al. 2018 [73]	Human WJ-MSCs	BPD	TSG-6	Decreased proinflammatory cytokines IL- 6, TNF-α, and IL-1β and cell death
	Wang et at. 2019 [92]	Human breastmilk	NEC	peptides derived from protein domain regions of lactotransferrin (LTF) and lactadherin (MFGE8)	LTF: stimulated intestinal cell proliferation MFGE8: promoted neural stem cell proliferation and migration, regeneration of injured intestinal mucosa by accelerating migration and proliferation through protein kinase C-dependent pathway
	Gussenhoven et al. 2019 [33]	Human BM-MSCs	HIE	Annexin A1	Formyl peptide receptor signaling promoting cytoskeletal stability, enhancing tight junction formation, and regulating BBB
	Kumar et al. 2019 [58]	Human P-MSCs	SB	Galectin 1	Involved in the adhesion of exosomes to cells
Nucleic acids	Lee et al. 2012 [72]	Mouse BM-MSCs	BPD	miRNA-16, miRNA-21, let7b pre-miRNA	Suppressed STAT3 and miR-17 microRNA superfamily, increased miR-204
	Joerger-Messerli et al. 2018 [37]	Human WJ-MSCs	HIE	let-7-5p miR	Suppressed caspase 3 involved in apoptosis
	Xu et al. 2019 [44]	BV2 microglial cells	ROP	miR-24-3p	Inhibited the inositol-requiring enzyme 1a (IRE1a)-X-box binding protein 1 (XBP1) cascade that contributes to apoptosis

Abbreviations: BM-MSCs, bone marrow mesenchymal stem cells; ROP, retinopathy of prematurity; BPD, bronchopulmonary dysplasia; VEGF, vascular endothelial growth factor; UCB-MSCs, umbilical cord blood-derived mesenchymal stem cells; WJ-MSCs umbilical cord Wharton’s jelly mesenchymal stem cells; IL-1β, interleukin 1 beta; IL-1α, interleukin 1 alpha; IL-6, interleukin 6; TNF-α, tumor necrosis factor alpha; TSG-6, tumor necrosis factor alpha-stimulated gene-6; NEC, necrotizing enterocolitis; LTF, lactotransferrin; MFGE8, lactadherin; HIE, hypoxic ischemic encephalopathy; BBB, blood–brain barrier; P-MSCs, placental mesenchymal stem cells; SB, spina bifida; STAT3, signal transducer and activator of transcription 3; miR, microRNA.
